# A Systematic Review and Meta-Analysis of Bovine Pestivirus Prevalence and Associated Risk Factors in Latin America

**DOI:** 10.3390/pathogens14060530

**Published:** 2025-05-26

**Authors:** Blanca Lisseth Guzmán Barragán, Isac Roman, Yessica Lorena Guzmán, Fernando Vicosa Bauermann

**Affiliations:** 1 Department of Veterinary Pathobiology, College of Veterinary Medicine, Oklahoma State University, 250 McElroy Hall, Stillwater, OK 74078, USA; 2 Department of Veterinary Medicine, Universidade Federal de Viçosa, Viçosa 36570-900, MG, Brazil

**Keywords:** BVDV, HoBi-like, meta-analyses, prevalence, risk factor

## Abstract

Bovine pestiviruses, namely bovine viral diarrhea virus (BVDV) and HoBi-like pestiviruses (HoBiPevs), are endemic viruses in Latin America, and the disease causes significant losses in the agricultural sector. The present review aims to perform a systematic assessment and meta-analysis of the prevalence of bovine pestiviruses in Latin America and their risk factors. Notable heterogeneity was observed in the analyzed groups, with significant prevalence variations based on age and country. However, no differences were found between temporal trends, production systems, or models. Identified risk factors included age, breed, location, reproductive practices, animal purchase, farm management, and biosecurity measures. This systematic review and meta-analysis of BVDV in Latin America provides critical insights to inform decision-making and strategic actions for disease control in the region. The high serological prevalence of bovine pestivirus across Latin America underscores the urgent need for standardized surveillance programs, biosecurity reinforcement, and targeted vaccination strategies. The presence of HoBiPev further complicates current diagnostic and control measures. Future research should focus on disease transmission dynamics, economic impact assessments, and the effectiveness of intervention programs tailored to the region’s diverse livestock production systems.

## 1. Introduction

Bovine pestiviruses, which include bovine viral diarrhea virus type 1 (BVDV-1), BVDV-2, and HoBi-like pestiviruses (HoBiPev), are endemic in Latin America and are widely distributed worldwide, exhibiting varying levels of prevalence in different regions [1]. Bovine pestiviruses belong to the *Pestivirus* genus of the *Flaviviridae* family and consist of enveloped positive-sense single-stranded RNA viruses [2]. BVDV-1, BVDV-2, and HoBiPev are, respectively, classified into *Pestivirus bovis*, *Pestivirus tauri*, and *Pestivirus brazilense* species [3]. Pestiviruses have two biotypes based on their effects on cultured cells: non-cytopathogenic and cytopathogenic [4]. In the Americas, based on genetic diversity studies, evidence suggests that BVDV-1 and BVDV-2 have been circulating since the 1670s [5]. More recently, HoBiPev has emerged and appears to be disseminated in many regions of the world, especially in South America [5,6]. Bovine pestiviruses primarily spread and persist in cattle populations through persistently infected (PI) animals, serving as the main reservoir. The virus has the ability to cross the placental barrier, and during the first trimester of gestation, the fetal infection may lead to the generation of the PI calves [7]. BVDV infection can also result in abortion and congenital anomalies [8]. In addition, BVDV also inflicts significant economic losses due to respiratory disease, mortality, and early culling [9,10]. Further, acute pestivirus infection may also lead to transient immunosuppression, increasing the incidence of secondary diseases [8].

The economic losses caused by the disease were described to range from USD 0.50 to USD 678.80 associated with mortality, morbidity, premature culling, stillbirths, abortion, and reinfection [11]. Strategies for bovine pestivirus control, such as the elimination of PI animals and vaccination, have been implemented in some countries in Europe [12,13] and North America [14]. However, these strategies are not widely adopted in Latin America [15]. The Latin American region is a strategic area for the world, located on the American continent. In this study, Latin America refers to countries in the Americas where Romance languages, primarily Spanish and Portuguese, are predominantly spoken. This includes both South and Central American nations as well as North America (Mexico). This broader regional framing allows for a more comprehensive and culturally coherent analysis of ruminant pestivirus prevalence and associated risk factors. It comprises 20 countries and accounts for 23% of global beef production and 11% of global milk production [16]. It has several countries with significant bovine production, including Brazil, Argentina, Mexico, Colombia, Paraguay, and Uruguay. Livestock farming in the region significantly contributes to the gross domestic product of the countries. In fact, Brazil has the largest commercial cattle population in the world.

The region also has growth potential for the livestock industry due to extensive territories, favorable weather, and abundant resources. Between 2000 and 2017, meat exports from Latin America increased by 29.8%, and milk exports increased by 35.7% [17]. However, these countries face great challenges related to herd health issues and production costs [16]. For instance, a study in Brazil reported that BVDV-related losses due to mortality and morbidity were estimated to be between USD 14,334.00 and USD 16,315.40 per 10,000 cattle [18].

Studies have estimated the prevalence of bovine pestiviruses worldwide [19,20,21]. Despite numerous global meta-analyses on BVDV, Latin America remains underrepresented in epidemiological studies. Given the region’s significance in global cattle production, understanding the disease’s prevalence and risk factors is essential for targeted control measures. This systematic review adds value by synthesizing previously fragmented, underrepresented, and often inaccessible Latin American data on bovine pestiviruses. Through exhaustive multilingual searches and the inclusion of local studies, it provides a more comprehensive regional epidemiological profile than global analyses.

## 2. Materials and Methods

The search strategy, screening protocols, and reporting for the systematic review and meta-analysis were conducted using the methodology Preferred Reporting Items for Systematic reviews and Meta-Analyses (PRISMA) [22].

### 2.1. Search Strategy

The objective of this systematic review and meta-analysis was to identify and analyze research articles on bovine pestiviruses in cattle across Latin American countries. A comprehensive literature search was conducted, selecting studies published in indexed journals, including PubMed, ScienceDirect, and Scopus. Eligible publications were those available in English, Spanish, or Portuguese and published between 2000 and 2024. The search strategy utilized specific keywords such as “Prevalence” OR “Frequency” followed by the disease name “BVDV” OR “Bovine Viral Diarrhea Virus” OR “HoBi-Like”, and the different countries of Latin America such as “Argentina”, “Bolivia”, “Brazil”, “Chile”, “Colombia”, “Costa Rica”, “Cuba”, “Dominican Republic”, “Ecuador”, “El Salvador”, “Guatemala”, “Haiti”, “Honduras”, “Mexico”, “Nicaragua”, “Panama”, “Paraguay”, “Peru”, “Uruguay”, and “Venezuela”. Considering the importance of PI animals, a search was carried out with “Persistent Infected” followed by the disease name “BVDV” OR “HoBi-Like”, and the different countries of Latin America. The criteria considered for the selection of studies are presented in Table 1.

Relevant articles were downloaded until 20 March 2024. Additionally, manual searches were conducted on Google Scholar. The search and screening process was conducted jointly by researchers proficient in Latin languages, BLGB, YLG, and FVB.

The following exclusion criteria were considered: (a) studies conducted outside of Latin America; (b) review studies; (c) studies lacking a summary or with inaccessible full text; (d) studies focusing on pestiviruses in non-bovine species; (e) case reports and case studies; (f) studies based solely on secondary data; (g) studies where disease epidemiology was not the primary objective (e.g., clinical or pathological studies); (h) studies involving animals previously identified as positive for bovine pestiviruses; (i) studies published outside the timeframe of 2000–2024; (j) samples that were analyzed in groups and subsequently not confirmed on an individual basis; (k) voluntary laboratory studies; (l) sampling in one or two herds or from one research center; (m) a lack of prevalence data; and (n) duplicated articles.

### 2.2. Quality Assessment

Considering the diversity of studies conducted in Latin America, an assessment of the quality and potential biases of the studies included in the meta-analysis was carried out, utilizing the Risk of Bias (ROB) assessment previously described [23]. The assessment consisted of a questionnaire comprising 11 questions related to study type, target population, epidemiological design, methodology, and analysis. Each question was scored based on whether it represented a high risk of bias (0 points), intermediate risk of bias (1 point), or low risk of bias (2 points). The total scores were calculated by summing up the scores for all questions. Two evaluators, IR and YLG, conducted the evaluation. An agreement analysis was carried out through the kappa coefficient. The questions were developed based on the criteria of the checklists proposed by the Critical Appraisal Skills Programme (CASP) for systematic review studies, available at https://casp-uk.net/casp-tools-checklists (accessed on 31 May 2024).

### 2.3. Data Analysis

Information such as title, objective, abstract, authors, the type of study, diagnostic methodology, sampling design, prevalence estimation, vaccination, and the year of publication was extracted from the selected articles and systematized in databases. The meta-analysis of the prevalence was conducted considering the prevalence at the animal and herd levels. A positive herd was defined as one containing at least one positive animal or, in the case of bulk milk samples, having at least one positive milk sample. The calculations of the prevalence meta-analyses and plots were constructed using Microsoft Office Excel Version 16.96 as previously described [24].

The statistical analysis included the calculation of the outcome, standard error, variance, study weight, and weighted effect size with the following equations.

$$\mathrm{Calculated}\;\mathrm{outcome}\;(\mathrm{ES})=\frac{Number\;of\;events}{Sample\;size}$$

$$\mathrm{Calculated}\;\mathrm{standard}\;\mathrm{error}\;(\mathrm{SE})=\frac{Square\;root\;of\;the\;outcomes}{Sample\;size}$$

Variance (Var) = SE^2^

Individual study weights (W) = 1/SE^2^

Heterogeneity was tested using the heterogeneity Cochran’s Q (Q), and I-squared statistic (I^2^).

Q = ∑ (W ∗ ES^2^) − [∑(W ∗ ES)]^2^/∑W

$$I^{2}\;\mathrm{index}=\frac{Q-degree\;of\;freedom\;(df)}{Q}\;*100$$

The random-effects model was used to calculate the summary effect, accounting for both between-study variability and within-study error [24]. In addition, subgroup analyses were performed to test the significant differences in the prevalence among countries, age, temporality, production system, and production model. To analyze the risk factors associated with bovine pestiviruses in Latin America, descriptive analyses were conducted, incorporating measures such as odds ratios, prevalence ratios, relative risks, results from univariate and multivariable analyses, logistic regression models, and statistical significance levels.

## 3. Results

**Figure 1:**
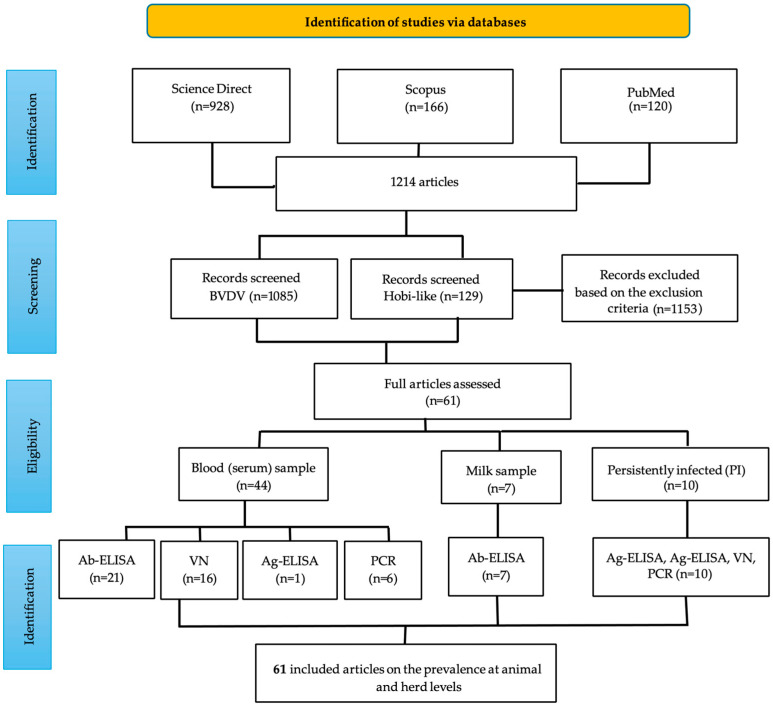
Flowchart of the selection process of the studies in the meta-analysis.

A comprehensive search retrieved 1214 studies related to bovine pestiviruses. Of these, 61 met the predefined inclusion criteria and were included in the meta-analysis (Figure 1). Only studies focusing on epidemiological aspects and conforming to the inclusion criteria were retained, although more than 400 studies addressed related topics such as immunology, clinical manifestations, reproduction, and virology. Additionally, approximately 490 studies, despite matching at least one search keyword, were unrelated to the study objectives, often due to partial or incidental keyword overlap, and were therefore excluded from further analysis. The studies included forty-four analyses using serum samples (Table 2), seven using milk samples (Table 3), and ten studies for the identification of PI animals (Table 4). The studies used different diagnostic tests such as Ab-ELISA, VN, Ag-ELISA, and RT-PCR. These studies were conducted in Argentina (two), Brazil (twenty-four), Chile (four), Colombia (ten), Costa Rica (one), Ecuador (two), Mexico (five), Peru (ten), and Uruguay (three) (Table 1). One study included animals from Mexico that crossed into the United States. The animals belonged to various production systems, including dairy, beef, intensive, and mixed.

**Figure 2:**
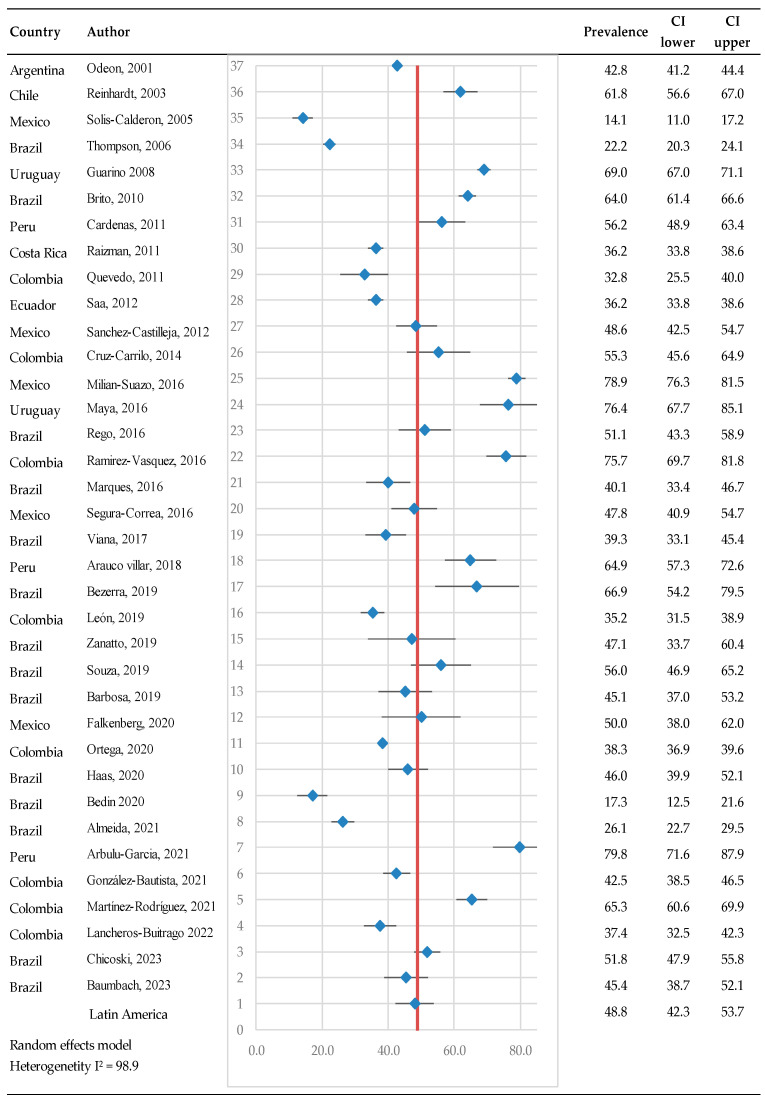
Meta-analysis of the prevalence of antibodies against bovine pestiviruses in serum (animal level) [25,26,27,28,29,30,31,32,33,34,35,36,37,38,39,40,41,42,43,45,46,47,48,49,50,51,52,53,54,55,56,57,58,59,60,61]. Each row represents a study or dataset, with prevalence percentages displayed alongside their 95% confidence intervals (CI). The blue diamonds represent the point estimates of prevalence, while the horizontal lines denote the corresponding confidence intervals.

The prevalence of bovine pestivirus antibodies in serum samples using a random-effects model was found to be 48.8% (95% CI: 42.3–53.7) (Figure 2). The seroprevalence included 36 studies at the animal level, 50,285 observations, and 25,324 events. The heterogeneity test results indicated Q = 322.4 and I^2^ = 98.9, suggesting high heterogeneity between studies. Significant statistical differences were observed between countries, with the highest prevalence reported in Peru at 79.8% [56], followed by Uruguay at 76% [29], and the lowest prevalence reported in Mexico at 13.9% [27]. Argentina had a prevalence of 42.8%, Ecuador 36%, Colombia 32.7% to 75.73%, Chile 61%, Costa Rica 36.2%, Mexico 13.9% to 78%, Peru 56.1% to 79.8%, and Uruguay 69.0% to 76.4%. However, there are great differences between regions of the same country. When considering individual countries, Brazil presented a prevalence ranging from 16.7% to 67.0%.

**Figure 3:**
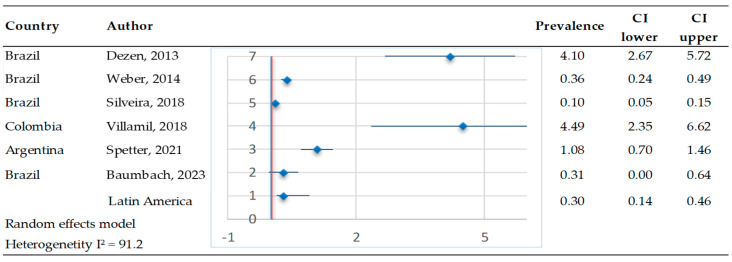
Meta-analysis of the prevalence of bovine pestivirus RNA in cattle [61,63,64,65,66,67]. Each row represents a study or dataset, with prevalence percentages displayed alongside their 95% confidence intervals (CI). The blue diamonds represent the point estimates of prevalence, while the horizontal lines denote the corresponding confidence intervals.

RT-PCR was used in six studies from Brazil, Colombia, and Argentina (Figure 3). The meta-analysis revealed a bovine pestivirus prevalence of 0.3% (95% CI 0.14–0.46%), involving 30,628 observations and 130 events. The heterogeneity test results indicated Q = 57.1 and I^2^ = 91.2. Differentiation between acutely and PI animals was not included in these studies.

**Figure 4:**
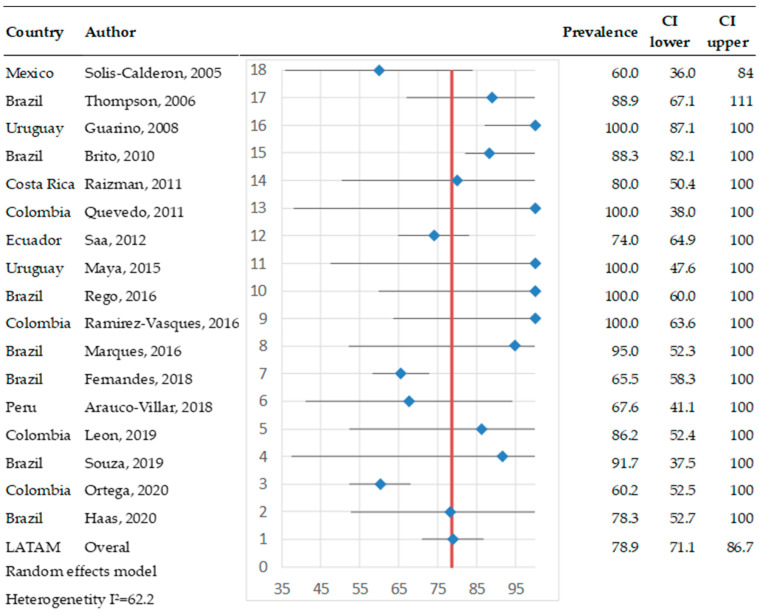
Meta-analysis of the prevalence of antibodies against bovine pestiviruses in serum (herd level) [27,28,29,30,32,33,34,38,39,40,41,44,45,47,49,52,53]. Each row represents a study or dataset, with prevalence percentages displayed alongside their 95% confidence intervals (CI). The blue diamonds represent the point estimates of prevalence, while the horizontal lines denote the corresponding confidence intervals.

The prevalence of antibodies at the herd level was more uniform compared to the prevalence observed in individual animals across Latin America. Seventeen studies provided data on herd-level prevalence, encompassing a total of 2695 herds. Among these, 2124 herds had at least one positive case. Five studies reported that 100% of herds contained at least one animal positive for bovine pestivirus, while the remaining studies indicated prevalence rates ranging between 60% and 90%. Overall, the herd-level prevalence was estimated at 78.9% (95% CI: 71.1–86.7%) (Figure 4). The heterogeneity test results showed Q = 42.3 and I^2^ = 62.2, indicating moderate heterogeneity across the studies.

**Figure 5:**
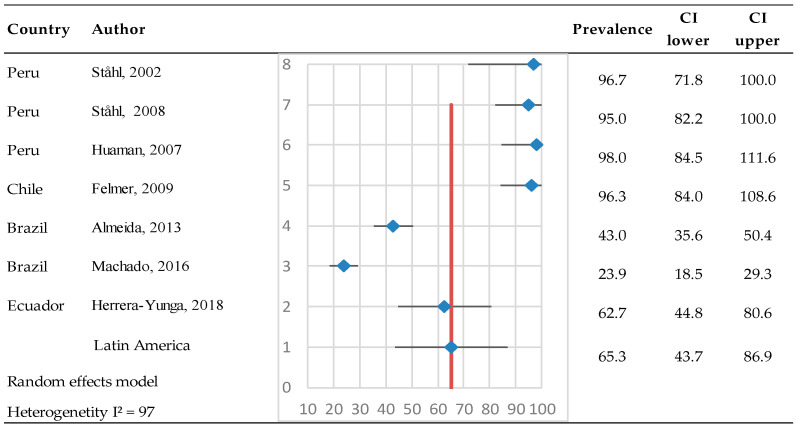
Meta-analysis of the prevalence of antibodies against bovine pestiviruses in milk (herd level) [68,69,70,71,72,73,74]. Each row represents a study or dataset, with prevalence percentages displayed alongside their 95% confidence intervals (CI). The blue diamonds represent the point estimates of prevalence, while the horizontal lines denote the corresponding confidence intervals.

Seven studies have been conducted to detect bovine pestiviruses in milk samples, primarily at the herd level, with three studies focusing on the individual animal level. At the herd level, a total of 1419 samples were evaluated, of which 955 tested positive. The estimated prevalence was 65.3% (95% CI: 43.7–86.9%) (Figure 5). The heterogeneity test results indicated Q = 258 and I^2^ = 97%, reflecting high heterogeneity.

**Figure 6:**
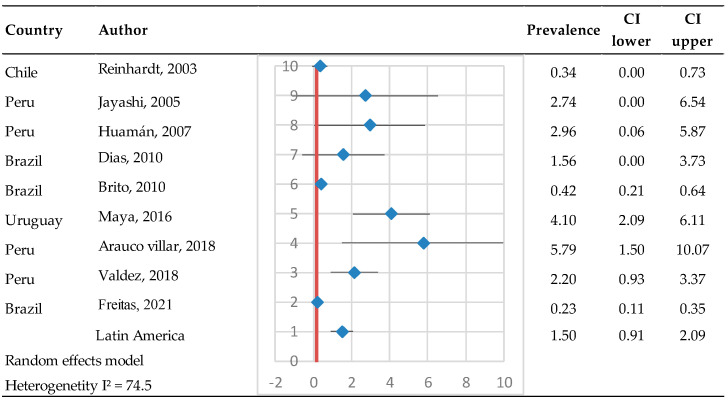
Prevalence of persistently infected (PI) animals [26,30,38,45,70,75,76,77,78]. Each row represents a study or dataset, with prevalence percentages displayed alongside their 95% confidence intervals (CI). The blue diamonds represent the point estimates of prevalence, while the horizontal lines denote the corresponding confidence intervals.

Various methodologies and diagnostic approaches were used to identify PI animals, including RT-PCR, Ab-ELISA, Ag-ELISA, and VN with various intervals between sampling to confirm the PI status. Studies have been conducted in Chile, Colombia, Brazil, and Peru (Figure 6). Most of the studies reported low frequencies of PI animals. The prevalence of PI animals was 1.5% in Latin America (95% IC 0.91–2.09%), involving a total of 5816 evaluated animals, with a total of 61 PI animals identified. The heterogeneity test results showed Q = 31.3 and I^2^ = 74.5, indicating high heterogeneity between studies.

Subgroup evaluation was conducted in serological studies at the animal level to observe the differences between the prevalences by age, countries, and the type of herd (Table 5). The analysis by age revealed differences in prevalence between animals under one year old, at 39.4%, and adult animals, at 55.1%. The prevalence by country shows high prevalence rates in Uruguay and Peru, while prevalence was low in Mexico. However, it is important to note that some countries have a more significant number of studies and better territorial coverage. No significant differences were observed in prevalence based on temporality, herd type, or production model.

The study of risk factors in Latin America was carried out using different methodologies and target variables. An association of the disease with geographical location was present in five studies and an association with increased animal age was present in four studies (Table 6). However, several studies addressed breed and production systems as risk factors for large and dense herds (seven), and the introduction, exchange, and purchase of animals was also a factor reported in several studies (nine). According to the reproduction model, both natural reproduction (five) practices and artificial insemination were reported as risk factors (five). Many different biosafety variables were related to the disease. However, no common variables were identified. Studies identified associations with coinfections, such as *Neospora caninum*, bovine herpesvirus type 1 (BoHV-1), mastitis, *Leptospira* spp., bovine leukemia virus (BLV), and parainfluenza virus 3 (PI-3), and with symptoms such as abortion, fever, reproductive problems, and mortality.

The quality and potential biases of all studies were evaluated. Notably, the assessment revealed differences among the studies. The recent studies received higher scores, reflecting improvements in methodologies. The results are presented in Supplementary Information. A Cohen’s value of 0.92 was observed, indicating a substantial agreement between the two researchers. Cohen’s kappa values for individual questions also demonstrated substantial agreement, except for questions 2 (0.6724) and 3 (0.7759), which showed moderate agreement, specifically related to sampling.

## 4. Discussion

This systematic review and meta-analysis aimed to estimate the prevalence of bovine pestiviruses in cattle across Latin America. While global reviews provide valuable macro-level insights, regional analyses such as the present review are essential to uncover epidemiological particularities, address locally relevant risk factors, and support the development of targeted, evidence-based control programs in Latin America. [19,20,21]. A comprehensive literature search on pestiviruses was conducted, identifying studies that employed diverse methodological approaches, diagnostic criteria, sampling strategies, and languages. Including a broad range of articles was intended to ensure robust regional representation. However, the limited number of studies from certain countries reduced the overall representativeness. Although a substantial number of studies on bovine pestiviruses were identified in the region, many were excluded due to not meeting the standardized epidemiological criteria necessary to ensure methodological rigor and comparability across studies.

The antibody prevalence in Latin America was 48.8%, which is slightly higher compared to the global prevalence of 43% reported by Werid [19] and Su [20] 42.7%. A study focusing on low- and middle-income countries reported a global prevalence of 66.3%. However, it found a prevalence of 45.2% for Latin America, which is slightly lower than observed in our study [79]. Both values from Latin America are higher than those reported for Sub-Saharan Africa (39.5%) and Asia (21.6%), but lower than the prevalence reported for the Middle East (49.9%) [79].

High heterogeneity was noted, aligning with previous reports [19,20,79]. However, our findings revealed significant disparities in serological prevalence rates across territories and countries, with values ranging from 6.3% to 79%. These differences were even more pronounced within different territories of the same country. Several authors have reported that certain territories, cities, districts, states, rural areas, and clusters were associated with an increased prevalence of the disease [42,44,47,60]. Such variations may be attributed to factors such as the concentration of animals in areas of higher productivity, geographic diversity, different vaccination practices, trade networks, animal management, or other epidemiological characteristics, including circulating viral species or strains. Notably, one report from Brazil distinguished between the prevalence of BVDV-1, BVDV-2, and the HoBiPev species and demonstrated high seroprevalence against HoBiPev [61]. Also in Brazil, several studies have demonstrated the significant prevalence of HoBiPev as well as the diversity of BVDV subtypes [64,80,81,82,83,84]. HoBiPev is not restricted to Brazil, as reports have demonstrated circulation also in Argentina [85,86].

The detection of HoBi-like pestiviruses (HoBiPev) in both Brazil and Argentina presents a critical challenge for pestivirus control across Latin America. Unlike classical BVDV-1 and BVDV-2, most commercially available diagnostics and vaccines were not designed to detect or protect against HoBiPev, raising concerns about underdiagnosis and vaccine escape. To address this gap, regional laboratories should implement multi-target RT-PCR protocols capable of distinguishing HoBiPev from other pestiviruses. Concurrently, the development of updated or trivalent vaccines incorporating HoBiPev antigens should be prioritized, particularly for regions with documented circulation. Official pestivirus surveillance and control programs must also be updated to include HoBiPev as a distinct epidemiological and regulatory category to ensure effective mitigation.

Overall, the prevalence at the herd level was determined to be 78.9%, and 65.3% in milk samples, which is considered high. This may be related to the lack of official control strategies and systematic use of vaccination in the region. At the herd level, no differences were found in prevalence based on the type of production and model, but some practices, such as mechanical milking, were reported as an associated risk factor [52,55]. Studies have shown that the prevalence of the disease is higher in dairy cattle compared to extensive systems [20,87]. In Latin America, dual-purpose production is frequently employed, alternating between extensive and semi-extensive production. Therefore, it was not possible to identify significant differences between the systems.

On the other hand, according to the risk analysis from several studies in Latin America, large herds in large areas with high population density were associated with the disease [34,41,42,57]. A meta-analysis conducted in Europe reported the association of large herds with the disease. However, it also identified studies in which small herds were associated with the disease [87]. Large herds have a greater probability of retaining pregnant animals and a more significant number of animals being purchased [87]. Conversely, smaller herds often lack systematic biosecurity measures, which may increase their vulnerability to disease introduction and spread.

Limited population-level prevalence studies using molecular techniques were observed. The identified studies reported prevalence rates ranging from 0.31% to 4.9% by RT-PCR, which are lower than those reported by global studies, which found a prevalence of 5% using antigen-based detection and 8% using nucleic acid techniques [19]. Persistently infected (PI) animals are the principal reservoirs of pestiviruses within herds, making their accurate identification a critical component of disease control. Differentiating PI animals from transiently infected individuals is essential for effective surveillance and management strategies [21]. A variety of methodologies were used to identify PI animals in Latin America, which complicates efforts to estimate their prevalence at the regional level. In several studies, persistent infection was assumed in animals that were initially negative for pestivirus antibodies and subsequently tested positive for antigen or viral RNA by RT-PCR. While it is true that PI animals are typically seronegative, a definitive diagnosis requires confirmation through two consecutive positive results obtained by virus isolation, antigen detection, or RT-PCR, with samples collected at least three weeks apart. This underscores the need for standardized approaches to PI detection.

The prevalence information disaggregated by age, and the recurring studies that identify age as a risk factor, show that the older the age, the greater the number of seropositive animals [40,57,59,60]. Age has consistently emerged as a significant risk factor in global studies, with older cattle more likely to exhibit long-term antibody responses due to prior vaccination, immunological maturity, and cumulative or repeated exposure to pestiviruses [1,9,88].

A relevant risk factor in Latin America, reported in seven studies, was the introduction or purchase of animals, which includes animal exchange and the participation of animals in livestock shows [27,33,40,41,42,46,57]. Many studies have confirmed that the purchase of animals is one of the most important factors [87,89]. Quantitative evaluation models have confirmed the association between animal movements, including purchasing or introducing livestock, raising replacement heifers off-site, and exhibiting livestock in competitions, and the risk of disease transmission. Benavides [90] reported that the movements of animals on farms increase the probability of the occurrence of the disease by 12%. In the Netherlands, studies on the efficiency of the control and eradication program have shown that testing purchased animals helped reduce the introduction of the virus [91]. Therefore, monitoring and limiting animal movements in herds can serve as a cost-effective strategy in Latin America.

Reproduction practices were a factor widely explored. However, it was a controversial aspect, as both artificial insemination [34,41,57] and the use of natural mating were identified as risk factors [33,40,45]. Contradictory reports related to reproduction can be due to variables that are associated with other procedures, such as adequate collections, the quality of the semen, and semen testing methods, as well as the origin of the bulls. In this study, for example, we found that the shared use of breeding bulls, a common practice in Latin America, was a relevant factor. In Argentina, an outbreak was reported that was associated with the presence of BVDV in semen [92]. This aspect has also been reported in several studies worldwide [93,94,95].

In Latin America, several farming practices have been studied and linked to the disease, including pasture leasing, weaning, natural breeding, proximity to neighboring cattle farms, colostrum feeding, feed management, the presence of rats, and needle reuse. These associations reflect the diverse range of farming practices used across the region [34,39,41,45,52,55,57,58]. However, key biosecurity measures such as quarantine, screening diagnosis, input control, and isolation have been rarely studied. This is particularly concerning given BVDV’s environmental resilience. The virus can persist for several hours to days in moist organic material under cool and dark conditions [96]. These characteristics highlight the importance of implementing and further investigating biosecurity practices. Evidence supports their effectiveness in preventing viral reintroduction [87], and studies have also reported economic benefits associated with their adoption [97,98].

Additionally, other infectious diseases, including *Neospora caninum*, bovine herpesvirus type 1 (BoHV-1), mastitis, *Leptospira* spp., bovine leukemia virus, and parainfluenza virus type 3 (PI-3), have been identified as risk factors [52,53,55,57,89]. Pestiviruses are frequently associated with respiratory diseases [33,39,41,52,55], likely due to their capacity to induce transient immunosuppression, which may facilitate secondary infections [99]. The authors state that primary infections may cause immunosuppression, increasing the likelihood of respiratory illness. Studies have reported symptoms associated with the disease, including abortion, fever, reproductive problems, and mortality. The disease has a brief acute symptomatic phase; however, some authors have observed more respiratory symptoms associated with bovine viral diarrhea virus (BVDV) than reproductive or digestive symptoms [19].

Several studies have identified breeds such as Holstein, Creole, Normande, and crossbreeds as potential risk factors for respiratory diseases in cattle. However, these associations have not been consistently observed across all research, suggesting variability depending on study context or population [34,57,58]. It is important to note the diversity of breeds used in the region. While the heritability of susceptibility to respiratory diseases appears to be low, breed differences have been noted. The breed has been a contentious risk factor, as it can be categorized based on breed usage [100]. Seasonal analysis was not conducted for winter and summer, as some tropical countries lack distinct seasons, and in others, seasonal variations are less pronounced. However, a study identified high altitudes as a risk factor, likely related to colder temperatures [34].

Despite the comprehensive scope of this review, several limitations must be acknowledged. First, substantial heterogeneity in study design strategies, sampling, and diagnostic protocols across studies posed challenges for statistical comparability and synthesis. Second, the inconsistent application of confirmatory testing, particularly for the identification of PI animals, may have contributed to misclassification. Third, the lack of longitudinal studies and time-series data limited our ability to evaluate trends in pestivirus prevalence over time. To strengthen the epidemiological evidence base in the region, future research should prioritize methodological standardization, consistent diagnostic criteria, and the development of multi-year surveillance frameworks.

## 5. Conclusions

This systematic review and meta-analysis reveal the significant prevalence and complex epidemiology of ruminant pestiviruses in Latin America. Prevalence rates vary widely across the region, shaped by a multifactorial interplay of biological, management, and infrastructural factors. Countries with large, intensive cattle industries, such as Brazil and Argentina, face higher transmission risks due to greater animal densities and frequent animal movements. In contrast, smaller-scale systems in parts of Central America may limit direct transmission but struggle with low diagnostic coverage and delayed disease detection. Differences in veterinary infrastructure, the availability of diagnostic tools, surveillance intensity, and informal cross-border trade further contribute to regional heterogeneity.

Effective pestivirus management in Latin America requires more than generalized regulatory strategies. It demands targeted interventions, including the implementation of mandatory PI detection and removal using standardized assays, consistent vaccination protocols to address emerging strains, strengthened animal movement controls, and sustained investment in diagnostic infrastructure and farmer education. Tailoring these measures to the socio-economic and production realities of each country is essential for reducing the pestivirus burden and improving animal health outcomes across the region.

Vaccination coverage also represents a critical challenge. Although vaccines are available in some countries, inconsistencies in uptake, cold chain maintenance, and strain matching, particularly for HoBi-like pestiviruses, compromise effectiveness [101,102]. The identification of HoBi-like pestiviruses (HoBiPev) in Brazil and Argentina adds complexity to pestivirus control, as current diagnostics and vaccines were primarily developed for classical BVDV strains. The genetic differences between HoBiPev and BVDV necessitate adaptations in surveillance, diagnostic tools, and vaccination programs to ensure accurate detection and effective disease control.

To operationalize these strategies, region-specific policy interventions are needed. Countries with high prevalence and intensive production systems, such as Brazil and Argentina, could benefit from piloting PI animal detection and removal programs. Such programs should be coupled with robust traceability and quarantine systems. For countries with fewer resources or smaller herd sizes, scalable solutions, such as subsidized testing for replacement heifers, targeted vaccination campaigns, and mobile diagnostic services, can serve as effective entry points. Additionally, integrating BVDV-free certification into livestock trade protocols may incentivize compliance and enhance disease transparency across national and cross-border markets.

## Figures and Tables

**Table 1:** Criteria employed for the selection of studies for the meta-analysis.

Category	Meta-Analysis
**Prevalence study**	Only studies that included data on the prevalence or the frequency of positive animals were considered. This was calculated using the formula (positive animals/sample population ∗ 100) at the animal level. Prevalence by herd was assessed by calculating the proportion of positive herds, defined as those containing at least one positive animal, divided by the total number of herds studied.
**Virus Species**	Studies that included different viral species of bovine pestiviruses: BVDV-1, BVDV-2, and HoBiPev.
**Animals Species**	Bovine (cattle).
**Country**	Latin American countries included Argentina, Bolivia, Brazil, Chile, Colombia, Costa Rica, Cuba, Dominican Republic, Ecuador, El Salvador, Guatemala, Haiti, Honduras, Mexico, Nicaragua, Panama, Paraguay, Uruguay, and Venezuela.
**Period**	Studies published between January 2000 and March 2024 were considered.
**Herd and Production System**	Studies including more than three herds were considered. Given the diverse production systems in Latin America, animals from all production types, including beef, mixed, extensive, and unspecified, were included
**Age**	Studies with animals of mixed ages were included, as well as those specifically with animals older than 12 months, 24 months, 36 months, and various ranges over 1 year of age. Studies exclusively with animals under one year of age were excluded from the serological evaluation.
**Sex**	Both male and female animals were included, along with groups consisting exclusively of males or females.
**Vaccination**	The serological evaluation included samples from non-vaccinated animals as well as samples from animals that were part of official vaccination programs for non-respiratory diseases. For the viral detection studies, samples from both vaccinated and unvaccinated animals were included.
**Sample Material**	For serological studies, serum and milk samples were included. For viral studies, blood, serum, milk, biopsy, nasal swab, and tissue samples were included. Samples from abortions and rectal swabs were not included.
**Sample Calculation and Sampling**	Studies that calculated the number of samples and provided information on the target population size were included. Various sampling methods, such as randomized sampling, stratified sampling, and convenience sampling, were considered. Samples obtained from official vaccination programs, non-captive animals, and animals in slaughterhouses were included. Studies that did not provide specific information were categorized as non-specific. Pooled serum sampling was included only if individual samples were later confirmed. Bulk milk samples were included.
**Diagnostic Method**	Serological studies included antibody detection ELISA (Ab-ELISA) and virus neutralization methods. Pathogen detection studies utilized antigen detection ELISA (Ag-ELISA), RT-PCR, and virus isolation assays.
**Persistently Infected**	Studies that identified PI animals by various methodologies were considered. Studies were included if two diagnostic tests, such as Ab-ELISA, Ag-ELISA, or RT-PCR, were performed. Blood and ear notch samples, as well as animals of different ages, were included.

**Table 2:** Studies evaluating the prevalence of bovine pestiviruses in bovine samples from Latin America. Diagnostic tests used included antibody detection enzyme-linked immunosorbent assay-based antibodies (Ab-ELISA), antigen detection ELISA (Ag-ELISA), RT-PCR, and virus neutralization (VN). * Not available.

Country	Sample Collection	Samples/Herds	Animal Age And Herd Type	Sampling	Diagnostic Test	Prevalence at Animal and Herd Level
Serology antibody						
Argentina [25]	NA *	6510/27	6–12 months, 1–2 years, and greater than 2 years	Random	Ab-ELISA and VN	Animal 42.8%
Chile [26]	NA	878/9	NA	Convenience	Ab-ELISA	Animal 61.8%
Mexico [27]	2001–2002	560/40	Semi-intensive beef farms	Random	Ab-ELISA	Animal 14% Herd 60%
Brazil [28]	2000	2343/72	0–6 months, 7–18 months, 19–30 months, and over 30 months/extensive	Random	VN	Animal 22.2% Herd 88.9%
Uruguay [29]	2000–2001	6358/230	Bulls over 2 years, cows over 3 years of age, and replacement heifers	Random	Ab-ELISA	Animal 69% Herd 100%
Brazil [30]	2002	3533/888	Cows older than 24 months	Random	VN	Animal 64% Herd 88.3%
Peru [31]	NA	406/114	Over 6 months	Random	VN	Animal 56.2%
Costa Rica [32]	2008	496/35	Over 12 months	Convenience	VN	Animal 36.2% Herd 80%
Colombia [33]	2011	238/10	Cow/extensive	Random	Ab-ELISA	Animal 32.8% Herd 100%
Ecuador [34]	2008–2009	2367/346	Dairy and dual-purpose dairy–beef	Random	Ab-ELISA	Animal 36.2% Herd 74%
Mexico [35]	NA	500/10	NA	Random	Ab-ELISA	Animal 48.6%
Colombia [36]	NA	228/9	Heifers and pregnant cows	NA	VN	Animal 55.3%
Mexico [37]	2010–2012	4487/182	Intensive, dual-purpose dairy–beef	Stratified	Ab-ELISA	Animal 78.9%
Uruguay [38]	2014	390/14	6 months to 1 year old	NA	Ab-ELISA	Animal 76.4% Herd 100%
Brazil [39]	2013–2014	319/24	Over 24 months/beef and dairy	Convenience	VN	Animal 51.1% Herd 100%
Colombia [40]	2014	1003/24	Mainly dairy farms	Proportional	Ab-ELISA	Animal 75.7% Herd 100%
Brazil [41]	2013–2014	352/20	Milk, beef, and mixed	Random	Ab-ELISA	Animal 40.1% Herd 95%
Mexico [42]	2010–2011	385/57	Reproductive age	Random	Ab-ELISA	Animal 47.8%
Brazil [43]	2013	400/5	Cows over 24 months	Convenience	VN	Animal 39.3%
Brazil [44]	2012–2013	2443/476	Cows over 24 months	Random	VN	Herd 65.5%
Peru [45]	NA	425/37	Dairy	Convenience	Ab-ELISA	Animal 64.9% Herd 67.6%
Brazil [46]	NA	160/16	Beef cows over 24 months/semi-intensive	Random	Ab-ELISA	Animal 66.9%
Colombia [47]	2017	1000/29	7 months and 13 years/dual-purpose	Random	Ab-ELISA	Animal 35.2% Herd 86.2%
Brazil [48]	2014–2015	102/12	Cows over 24 months/dairy and breeding	Convenience	VN	Animal 47.1%
Brazil [49]	2017	257/12	Dairy and beef	Random	VN	Animal 56% Herd 91.7%
Brazil [50]	2013	264/20	Mixed breed dairy cows/extensive	Random	VN	Animal 45.1%
Mexico [51]	2017–2018	134/NA	NA	Convenience	VN	Animal 50%
Colombia [52]	2016–2018	8110/387	NA	Random	Ab-ELISA	Animal 38.3% Herd 60%
Brazil [53]	2013	476/46	Cows over 24 months	Random	VN	Animal 46% Herd 78.3%
Brazil [54]	2014	317/18	Over 1 year/dairy in semi-intensive and intensive systems	Convenience	VN	Animal 17%
Brazil [55]	2015	854/72	Dairy cows	Random	Ab-ELISA	Animal 26.1%
Peru [56]	2018	460/114	Over 4 months	Stratification	Ab-ELISA	Animal 79.8%
Colombia [57]	2019	1000/65	NA	Random	Ab-ELISA	Animal 42.5%
Colombia [58]	2016–2017	1157/46	Dual-purpose in extensive and semi-intensive systems	Random	Ab-ELISA	Animal 65.2%
Colombia [59]	NA	601/NA	Mainly dairy cows	Random	Ab-ELISA	Animal 37.4%
Brazil [60]	NA	1266/31	Males 8 to 32 months	Convenience	VN	Animal 51.8%
Brazil [61]	2014–2015	390/NA	6 and 24 months	Random	VN	Animal 45%
**Antigen detection**						
Chile [62]	NA	4998/150	Lactating cows	Random	Ag-ELISA	Animal 6.3%
**RT-PCR**						
Brazil [63]	NA	692/6	Female and male	Convenience	RT-PCR	Animal: 4.2%
Brazil [64]	NA	9078/346	6 to 12 months	Random	RT-PCR	Animal 0.4% Herd: 6.9%
Brazil [65]	2012–2013	16,621/569	Up to 24 months of age/predominantly beef	Random	RT-PCR	Animal 0.1% (HoBiPev)
Colombia [66]	2014	379/15	240–255 days of gestation	Random	RT-PCR	Animal 4.5%
Argentina [67]	2015–2019	2864/55	Beef and dairy cattle	BVDV control program	RT-PCR	Animal 1% Herd 20%
Brazil [61]	2014–2015	994/NA	6 and 24 months	Random	RT-PCR	Animal 0.3%

**Table 3:** Studies evaluating the prevalence of bovine pestiviruses in bovine milk samples from Latin America. Sample tested by antibody detection enzyme-linked immunosorbent assay-based (Ab-ELISA). * Not available.

Country	Study Year	Samples/Herds	Animal Age and Herd Type	Sampling	Diagnostic Test	Prevalence at Animal and Herd Level
Peru [68]	1998	60/60	NA*/dairy cows	Categorized	Ab-ELISA	Herd: 96%
Peru [69]	2003–2004	387/221	NA/dairy cows	Random	Ab-ELISA	Herd: 95%
Peru [70]	2004	204/204	6 to 24 months/dairy cows	Convenience	Ab-ELISA	Herd: 98%
Chile [71]	2004–2005	649/279	NA/dairy cows	Convenience	Ab-ELISA	Herd: 96%
Brazil [72]	2009	300/300	NA/dairy cows	Random	Ab-ELISA	Herd: 43%
Brazil [73]	2011	314/314	NA/dairy cows	Random	Ab-ELISA	Herd: 23.9%
Ecuador [74]	2015	394/75	Semi-intensive and extensive	Random	Ab-ELISA	Herd: 63.5%

**Table 4:** Studies evaluating the prevalence of PI calves in Latin America. Diagnostic tests used included Ab-ELISA, Ag-ELISA, RT-PCR, and VN. * Not available.

Country	Study Year	Samples/Herds	Animal Age and Herd Type	Sampling	Screening Test	Time Between Sampling and Second Test	Prevalence
Chile [26]	NA *	878/9	Over 6 months	Convenience	Ab-ELISA	3 weeks. Antibody-negative samples tested by Ag-ELISA	Animal 0.3%
Peru [75]	NA	3/1	6 to 12 months	Convenience	Ab-ELISA	30 days. Ag-ELISA	Animal 2.7%
Peru [70]	NA	286/57	Female 6 to 24 months	Convenience	Ab-ELISA	NA. Antibody-negative samples tested by Ag-ELISA	Animal 2.9%
Brazil [76]	NA	512/26	6–12 months	Random	VN	30 days. RT-PCR	Animal 3.1%
Brazil [63]	NA	692/6	Female and males	Convenience	RT-PCR	4 months. RT-PCR	Animal 0.4%
Uruguay [38]	2014	390/14	6 months to 1 year	NA	Ab-ELISA	NA. Antibody-negative samples tested by Ag-ELISA or RT-PCR	Animal 4.1%
Peru [45]	NA	121/37	3 to 15 months	Convenience	Ab-ELISA	NA. Ag-ELISA	Animal 5.8%
Peru [77]	NA	1135/NA	NA	Convenience	Ab-ELISA	30 days. Antibody-negative samples tested by Ag-ELISA	Animal 2.2%
Brazil [78]	2015 2018	6465/40	Cows under 2 years	Random	Ear notch test, Ag-ELISA	NA	Animal 0.2%

**Table 5:** Prevalence by demographic characteristics and production systems in Latin America. The table includes confidence intervals (CI), heterogeneity measured by Cochran’s Q (Q), and the I-squared statistic (I^2^) to assess variability across studies. Country acronyms: Argentina (AR), Colombia (COL), Chile (CL), Ecuador (ECU), Peru (PE), Mexico (MX), Brazil (BRA), Uruguay (UR), and Costa Rica (CR).

Category	Studies	Positive Samples	Tested Samples	Low	High	Country	Prevalence	CI	Q	I^2^
**Country**										
Brazil	13	5034	11,015	16.7%	67.1%	BR	45.4%	45.2–45.5%	427.2	92.7
Colombia	8	5669	13,125	32.7%	75.73%	COL	47.7%	39.1–56.3%	270.5	97.4
Peru	3	871	1291	56.1%	79.78%	PE	66.8%	53.4–80.1%	18.0	88.9
Mexico	5	4113	6066	14%	79%	MX	47.8%	44.6–50.9%	983	99
Uruguay	2	4686	6748	69%	76%	URG	71.4%	64.3–77.6%	2.6	62.2
**Age**										
Up to 1 year	8	1839	5274	17%	64%	BR, COL, AR, PE	39.4%	30.1–47.8%	58	87
1 to 2 years	6	950	1898	31%	90%	BR, COL, AR, PE	53.2%	48.0–63.2%	41	88
2 to 3 years	3	194	396	40%	50%	COL, PU, MX	48.1%	37.3–58.8%	5.1	60
Over 3 years	7	2386	4019	17%	71%	BR, COL, AR, PE	55.1%	36.3–73.6%	413	98.5
**Period of Time**										
2000–2016	18	17,688	32,912	13%	78%	BR, COL, PE, MX, EC, UR, CR, CL	48.8%	38.5–57.4%	2526	99.3
2017–2023	18	7525	17,355	17%	80%	BR, COL, MX, PE	48.1%	42.0–54.1%	525	96.7
**Herd Type**										
Dairy	14	2553	5449	16.7%	75%	BR, COL, PE, MX	46.9%	36.0–55.9%	405	97
Beef	8	5545	9101	14%	67%	MX, UR, BR	43.7%	24.5–63%	878	99.2
Mixed	7	2755	1157	22%	70%	BR, ECU, COL	44.1%	34.3–53.9%	349	98.3
**Production Model**										
Extensive production	11	2950	6744	16.7%	79%	MX, BR, COL, PE	47.8%	35.0–61.3%	1030	98.6
Intensive production	6	999	2174	13.0%	76%	AR, CL, MX, PR, BR	52.7%	27.2–78.1%	173	98.8

**Table 6:** Descriptive analysis of the risk factors associated with the presence of BVDV in Latin America, 2000–2024.

Risk Factor	Description	BVDV
Geographical location	Specific territories (city, municipality, state, cluster or rural, and district) were associated with increased prevalence	[42,44,47,54,60]
	Altitude of the farm >2338 m	[34]
Age	Older than 1 year	[57,59,60]
	Older than 2 years	[57,59,60]
	Older than 3 years	[40,59,60]
	Older than 4 years	[57,59,60]
Breed	Holstein, Jersey, Creole, Normande, and crossbred animals	[34,54,57,58]
Herd	Large herd	[37,42,57,72]
	Farm size ≤ 120 hectares	[41]
	High animal density	[34,41]
Farm management	Extensive system	[37,49]
	Common sheds	[37]
	Not extensive breeding system	[45]
	Mixed age animals	[62]
	Mixed herds (dairy–beef)	[49,50]
	Herd with open system of the production	[62]
	Herd with open-closed system of the production	[45]
	Calving intervals (≥ 395 days)	[37]
	Sell milk to different industries	[73]
Reproduction	Artificial insemination	[34,41,57,72,74]
	Use of bull	[45,50,73]
	Sharing of bulls between farms	[40]
Introduction of animals or purchase of animals	Introduces animals to the herd from external sources	[27,33,40,42,46,57]
	Animal exchange	[41]
	Participation of the animals in livestock shows	[57]
Co-infection	Animals with BoHV-1 were correlated with the presence of BVDV	[52,53]
	*Neospora caninum*, *Leptospira* spp., and bovine leukemia virus infections were correlated with the presence of BVDV	[52]
	Mastitis was correlated with the presence of BVDV	[52,55]
	PI-3 was correlated with the presence of BVDV	[57]
Biosecurity	Re-use of needles	[58]
	Calving paddock	[50]
	No quarantine	[50,73]
	Presence of rodents	[45]
	Pasture leasing	[57,58]
	Burying dead animals on the farm	[52,58]
	Weaning age ≤ 60 days	[41]
	Natural breeding	[55]
	Use of mechanical milking	[50,52,55]
	Bordering cattle farms	[34,73]
	Colostrum not provided, consortium breeding	[39]
	Pelleted feed, or supplementation with molasses	[58]
Clinical signs	Abortion was correlated with the presence of BVDV	[33,52]
	Fever was correlated with the presence of BVDV	[52]
	Reproductive problems were correlated with the presence of BVDV	[55]
	Unknown diseases were correlated with the presence of BVDV	[39]
	Calf mortality > 5% was correlated with the presence of BVDV	[41]

## Data Availability

The original contributions presented in this study are included in the article/Supplementary Material. Further inquiries can be directed to the corresponding authors.

## Supplementary Materials

The following supporting information can be downloaded at: https://www.mdpi.com/article/10.3390/pathogens14060530/s1, Supplementary information—Risk of bias evaluation for the included studies.
