# Supplementary material for: A Systematic Review and Meta-Analysis of Bovine Pestivirus Prevalence and Associated Risk Factors in Latin America
Source: Pathogens. 2025 May 26;14(6):530. doi: 10.3390/pathogens14060530 (PMC12196217; doi:10.3390/pathogens14060530)
Supplement: Supplementary file 1 [file pathogens-14-00530-s001.zip › 5-25-25 pathogens-3571029-supplementary[90]-highlight.pdf]

Supplementary Information—Risk of Bias Evaluation for the Included Studies.

A total of 11 questions were included. The scoring system was as follows: high risk of bias = 0 points, intermediate risk = 1 point, and low risk of bias = 2 points. The total score represents the sum of all individual question scores and was used to determine the overall risk of bias for each study. The evaluation was independently conducted by two reviewers (IJR and YLG). Agreement between the evaluators was assessed using the kappa coefficient. The criteria for the assessment were based on the Critical Appraisal Skills Programme (CASP) checklists for systematic review studies.

**N.**	**Questions**	**High**	**Intermediate risk**	**Low**
**1**	Was there a clear statement on the aims of the research?			
**2**	Was the recruitment or Sampling method appropriate?			
**3**	Was the sampling frame a true or close representation of the target population?			
**4**	Was data collected in a way that addressed the research issue?			
**5**	Was Data Analysis Sufficiently rigorous?			
**6**	Was the Research Design appropriate?			
**7**	Was Ethical issues taken into consideration?			
**8**	Is there a clear statement of finding? (Outcome reporting and completeness)			
**9**	How Valuable is the research? (Contributions the study makes to existing knowledge)			
**10**	Any sponsorship Funding bias?			
**11**	Other sources of bias?			
**12**	**Total**			

Fonte: Chiocchia et al., 2021.

Results

Results of the risk of bias (ROB) assessment of the first evaluator.

Study	Question 1	Question 2	Question 3	Question 4	Question 5	Question 6	Question 7	Question 8	Question 9	Question 10	Question 11	Total
Odeon, 2001 [25]	2	1	2	2	2	2	0	2	2	2	2	19
Reinhardt, 2003 [26]	2	2	0	0	2	2	0	2	2	2	2	16
Solis-Calderon, 2005 [27]	2	2	2	2	2	2	0	2	2	2	2	20
Thompson, 2006 [28]	2	1	0	2	2	2	0	2	0	2	2	15
Guarino, 2008 [29]	2	0	2	2	2	2	0	2	2	2	2	18
Brito, 2010 [30]	2	2	2	2	2	2	0	2	2	2	2	20
Cardenas, 2011 [31]	2	0	0	2	2	2	0	2	2	2	2	16
Raizman, 2011 [32]	2	0	2	2	2	2	0	2	2	2	2	18
Quevedo, 2011 [33]	2	2	2	2	2	2	2	2	2	2	2	22
Saa, 2012 [34]	2	2	2	2	2	2	0	2	2	2	2	20
Sanchez Castilleja, 2012 [35]	2	0	0	0	1	1	0	2	2	2	2	12
Cruz-Carrillo, 2014 [36]	2	0	0	0	1	1	0	2	1	2	2	11
Milian Suoza, 2016 [37]	2	2	2	2	2	2	0	2	2	2	2	20
Maya, 2016 [38]	2	0	0	2	2	2	1	2	2	2	2	17
Rego, 2016 [39]	2	2	2	2	2	2	2	2	2	2	2	22
Ramirez-Vasquez, 2016 [40]	2	0	1	2	2	2	2	2	2	2	2	19
Marques, 2016 [41]	2	2	2	2	2	2	2	2	2	2	2	22
Segura-Correa, 2016 [42]	2	2	2	2	2	2	0	2	2	2	2	20
Viana, 2017 [43]	2	0	0	2	2	2	0	2	2	2	2	16

Fernandes, 2018 [44]	2	1	1	2	2	2	2	2	2	2	2	20
Arauco-Villar, 2018 [45]	2	2	2	2	2	2	0	2	2	2	2	20
Bezerra, 2019 [46]	2	2	2	2	2	2	2	2	2	2	2	22
León, 2019 [47]	2	2	2	2	2	2	2	2	2	2	2	22
Zanatto, 2019 [48]	2	0	0	2	2	1	0	2	1	2	2	14
Souza, 2019 [49]	2	0	2	2	2	2	2	2	2	2	2	20
Barbosa, 2019 [50]	2	0	1	2	2	2	0	2	0	2	2	15
Falkenberg, 2022 [51]	1	1	0	2	2	2	2	2	2	2	2	18
Ortega, 2020 [52]	2	2	2	2	2	2	2	2	2	2	2	22
Haas, 2020 [53]	2	0	0	2	2	2	2	2	2	2	2	18
Bedin, 2020 [54]	2	0	0	2	2	2	2	2	1	2	2	17
Almeida, 2021 [55]	2	2	2	2	2	2	2	2	2	2	2	22
Arbulú-García, 2021 [56]	2	2	2	2	2	2	0	2	2	2	2	20
González-Bautista, 2021 [57]	2	2	2	2	2	2	2	2	2	2	2	22
Martínez-Rodríguez 2021 [58]	2	2	2	2	2	2	2	2	2	2	2	22
Lancheros-Buitrago 2022 [59]	2	2	2	2	2	2	2	2	2	2	2	22
Chicoski, 2023 [60]	2	1	1	2	2	2	2	2	2	2	2	20
Baumbach, 2023 [61]	2	2	1	2	2	2	2	2	2	2	2	21
Antigen												
Alocilla, 2022 [62]	2	2	2	2	2	2	0	2	2	2	2	20
PCR												
Dezen, 2013 [63]	2	0	0	0	1	1	0	2	2	2	2	12
Weber, 2014 [64]	2	2	2	2	2	2	2	2	2	2	2	22

Silveira, 2018 [65]	2	2	2	2	2	2	2	2	2	2	2	22
Villamil, 2018 [66]	0	0	0	2	2	2	0	2	2	2	2	14
Spetter, 2021 [67]	2	2	2	2	2	2	2	2	2	2	2	22
Baumbach, 2023 [61]	2	2	1	2	2	2	2	2	2	2	2	21
Milk												
Ståhl, 2002 [68]	2	2	1	2	2	2	2	2	2	2	2	21
Ståhl, 2008 [69]	2	2	2	2	2	2	0	2	2	2	2	20
Huaman, 2007 [70]	2	2	2	2	1	1	0	2	2	2	2	18
Almeida, 2013 [72]	2	2	2	2	2	2	0	2	2	2	2	20
Machado, 2016 [73]	2	2	2	2	2	2	2	2	2	2	2	22
Herrera-Yunga, 2018 [74]	2	2	2	2	2	2	0	2	2	2	2	20
PI												
Reinhardt, 2003 [26]	2	2	0	0	2	2	0	2	2	2	2	16
Jayashi, 2005 [75]	2	0	0	0	2	2	0	2	2	2	2	14
Huamán, 2007 [70]	2	2	2	2	1	1	0	2	2	2	2	18
Dias, 2010 [76]	2	1	0	2	2	2	0	2	2	2	2	17
Brito, 2010 [30]	2	2	2	2	2	2	0	2	2	2	2	20
Maya, 2016 [38]	2	0	0	2	2	2	1	2	2	2	2	17
Arauco villar, 2018 [45]	2	2	2	2	2	2	0	2	2	2	2	20
Valdez, 2018 [77]	2	1	1	2	2	2	0	2	2	2	2	18
Freitas, 2021 [78]	2	2	1	2	2	2	2	2	2	2	2	21

Results of the risk of bias (ROB) assessment of the second evaluator.

Study	Question 1	Question 2	Question 3	Question 4	Question 5	Question 6	Question 7	Question 8	Question 9	Question 10	Question 11	Total
Odeon, 2001 [25]	2	2	0	2	2	2	0	2	2	1	2	17
Reinhardt, 2003 [26]	2	0	0	0	2	2	0	2	2	2	2	14
Solis-Calderon, 2005 [27]	2	2	2	2	2	2	0	2	2	2	2	20
Thompson, 2006 [28]	2	0	0	2	2	2	0	2	2	2	2	16
Guarino, 2008 [29]	2	1	2	2	2	2	0	2	2	2	2	19
Brito, 2010 [30]	2	2	2	2	2	2	0	2	2	2	2	20
Cardenas, 2011 [31]	2	2	0	2	2	2	0	2	2	2	2	18
Raizman, 2011 [32]	2	0	2	2	2	2	0	2	2	2	2	18
Quevedo, 2011 [33]	2	2	2	2	2	2	0	2	2	2	2	20
Saa, 2012 [34]	2	2	2	2	2	2	0	2	2	2	2	20
Sanchez Castilleja, 2012 [35]	2	0	0	0	1	1	0	2	2	2	2	12
Cruz-Carrillo, 2014 [36]	2	0	0	0	1	1	0	2	1	2	2	11
Milian Suoza, 2016 [37]	2	2	0	2	2	2	0	2	2	1	2	17
Maya, 2016 [38]	2	0	0	2	2	2	0	2	2	2	2	16
Rego, 2016 [39]	2	0	2	2	2	2	2	2	2	2	2	20
Ramirez-Vasquez, 2016 [40]	2	2	2	2	2	2	2	2	2	2	2	22
Marques, 2021 [41]	2	2	2	2	2	2	2	2	2	2	2	22
Segura-Correa, 2016 [42]	2	2	2	2	2	2	0	2	2	2	2	20

Viana, 2017 [43]	2	0	0	2	2	2	0	2	2	2	2	16
Fernandes, 2018 [44]	2	2	2	2	2	2	2	2	2	2	2	22
Arauco-Villar, 2018 [45]	2	0	2	2	2	2	0	2	2	2	2	18
Bezerra, 2019 [46]	2	2	1	2	2	2	2	2	2	2	2	21
León, 2019 [47]	2	2	2	2	2	2	2	2	2	2	2	22
Zanatto, 2019 [48]	2	0	0	2	2	1	0	2	2	2	2	15
Souza, 2019 [49]	2	1	2	2	2	2	2	2	2	2	2	21
Barbosa, 2019 [50]	2	0	0	2	2	2	0	2	1	2	2	15
Falkenberg, 2020 [51]	2	1	0	2	2	2	2	2	2	2	2	19
Ortega, 2020 [52]	2	2	2	2	2	2	2	2	2	2	2	22
Haas, 2020 [53]	2	0	0	2	2	2	2	2	2	2	2	18
Bedin, 2020 [54]	2	0	0	2	2	2	2	2	1	2	2	17
Almeida, 2021 [55]	2	2	2	2	2	2	2	2	2	2	2	22
Arbulú-García, 2021 [56]	2	2	2	2	2	2	0	2	2	2	2	20
González-Bautista, 2021 [57]	2	2	2	2	2	2	2	2	2	2	2	22
Martínez-Rodríguez 2021 [58]	2	2	2	2	2	2	2	2	2	2	2	22
Lancheros-Buitrago 2022 [59]	2	2	2	2	2	2	2	2	2	2	2	22
Chicoski 2023 [60]	2	0	2	2	2	2	2	2	2	2	2	20
Baumbach, 2023 [61]	2	2	0	2	2	2	2	2	2	2	2	20
Antigen												
Alocilla, 2022 [62]	2	2	2	2	2	2	0	2	2	2	2	20
PCR												

Dezen, 2013 [63]	2	2	0	0	1	1	0	2	2	2	2	14
Weber, 2014 [64]	2	2	2	2	2	2	2	2	2	2	2	22
Silveira, 2018 [65]	2	2	2	2	2	2	2	2	2	2	2	22
Villamil, 2018 [66]	2	0	0	0	2	1	0	2	2	2	2	13
Spetter, 2021 [67]	2	0	0	2	2	2	0	2	2	2	2	16
Baumbach, 2023 [61]	2	2	0	2	2	2	2	2	2	2	2	20
Milk												
Ståhl, 2002 [68]	2	2	2	2	2	2	0	2	2	2	2	20
Ståhl, 2008 [69]	2	2	2	2	2	2	0	2	2	2	2	20
Huaman, 2007	2	2	2	2	2	2	0	2	2	2	2	20
Almeida, 2013 [72]	2	2	2	2	2	2	0	2	2	2	2	20
Machado, 2016 [73]	2	2	2	2	2	2	2	2	2	2	2	22
Herrera-Yunga, 2018 [74]	2	2	2	2	2	2	0	2	2	2	2	20
PI												
Reinhardt, 2003 [26]	2	0	0	0	2	2	0	2	2	2	2	14
Jayashi, 2005 [75]	2	2	0	2	2	2	0	2	2	1	2	17
Huaman, 2007	2	2	2	2	2	2	0	2	2	2	2	20
Dias, 2010 [76]	2	2	0	2	2	2	0	2	2	2	2	18
Brito, 2010[30]	2	2	2	2	2	2	0	2	2	2	2	20
Maya, 2016 [38]	2	0	0	2	2	2	0	2	2	2	2	16
Araucovillar, 2018 [45]	2	0	2	2	2	2	0	2	2	2	2	18
Valdez, 2018 [77]	2	0	0	2	2	2	0	2	2	1	2	15
Freitas 2021 [78]	2	0	2	2	2	2	2	2	2	1	2	19

A Cohen's value of 0.92 was observed, indicating a substantial agreement between the two researchers. Cohen's kappa values for individual questions also demonstrated substantial agreement, except for questions 2 (0.6724) and 3 (0.7759), which showed moderate agreement, specifically related to sampling.

Cohen's kappa values analysis

Kappa	Low	Intermediate risk	High	
Low	515	14	6	
Intermediate risk	6	10	3	
High	13	9	62	0.9201

Cohen's kappa values analysis for question 1

Kappa	Low	Intermediate risk	High	
Low	57	1		
Intermediate risk				
High				0.9828

Cohen's kappa values analysis for question 2

Kappa	Low	Intermediate risk	High	
Low	28	3	4	
Intermediate risk	0	1	3	
High	7	3	10	0.6724

Cohen's kappa values analysis for question 3

Kappa	Low	Intermediate risk	High	
Low	29	5	0	
Intermediate risk	1	0	0	
High	3	4	16	0.7759

Cohen's kappa values analysis for question 4

Kappa	Low	Intermediate risk	High	
Low	52	0	0	
Intermediate risk	0	0	0	
High	1	0	5	0.9828

Cohen's kappa values analysis for question 5

Kappa	Low	Intermediate risk	High	
Low	53	2	0	
Intermediate risk	0	3	0	
High	0	0	0	0.9655

Cohen's kappa values analysis for question 6

Kappa	Low	Intermediate risk	High	
Low	52	2	0	
Intermediate risk	0	4	0	
High	0	0	0	0.9655

Cohen's kappa values analysis for question 7

Kappa	Low	Intermediate risk	High	
Low	22	0	0	
Intermediate risk	0	0	0	
High	3	2	31	0.9138

Cohen's kappa values analysis for question 8

Kappa	Low	Intermediate risk	High	
Low	58	0	0	
Intermediate risk	0	0	0	
High	0	0	0	1

Cohen's kappa values analysis for question 9

Kappa	Low	Intermediate risk	High	
Low	53	1	1	
Intermediate risk	0	2	1	
High	0	0	0	0.9483

Cohen's kappa values analysis for question 10

Kappa	Low	Intermediate risk	High	
Low	53	0	0	
Intermediate risk	0	5	0	
High	0	0	0	0.999

Cohen's kappa values analysis for question 11

Kappa	Low	Intermediate risk	High	
Low	58	0	0	
Intermediate risk	0	0	0	
High	0	0	0	1

Reference

Chiocchia, V.; Nikolakopoulou, A.; Higgins, J.P.T.; Page, M.J.; Papakonstantinou, T.; Cipriani, A.; Furukawa, T.A.; Siontis, G.C.M.; Egger, M.; Salanti, G. ROB-MEN: a tool to assess risk of bias due to missing evidence in network meta-analysis. *BMC Medicine*. **2021**, *19*, 1–13.
